# The Important Role of Lipid Raft-Mediated Attachment in the Infection of Cultured Cells by Coronavirus Infectious Bronchitis Virus *Beaudette* Strain

**DOI:** 10.1371/journal.pone.0170123

**Published:** 2017-01-12

**Authors:** Huichen Guo, Mei Huang, Quan Yuan, Yanquan Wei, Yuan Gao, Lejiao Mao, Lingjun Gu, Yong Wah Tan, Yanxin Zhong, Dingxiang Liu, Shiqi Sun

**Affiliations:** 1 State Key Laboratory of Veterinary Etiological Biology, Lanzhou Veterinary Research Institute, Chinese Academy of Agricultural Sciences, Xujiaping, Lanzhou, Gansu, The P.R. China; 2 School of Biological Sciences, Nanyang Technological University, Singapore, Singapore; 3 College of Animal Science, Yangtze University, Jingzhou, P.R. China; Sun Yat-Sen University, CHINA

## Abstract

Lipid raft is an important element for the cellular entry of some viruses, including coronavirus infectious bronchitis virus (IBV). However, the exact role of lipid rafts in the cellular membrane during the entry of IBV into host cells is still unknown. In this study, we biochemically fractionated IBV-infected cells via sucrose density gradient centrifugation after depleting plasma membrane cholesterol with methyl-β-cyclodextrin or Mevastatin. Our results demonstrated that unlike IBV non-structural proteins, IBV structural proteins co-localized with lipid raft marker caveolin-1. Infectivity assay results of Vero cells illustrated that the drug-induced disruption of lipid rafts significantly suppressed IBV infection. Further studies revealed that lipid rafts were not required for IBV genome replication or virion release at later stages. However, the drug-mediated depletion of lipid rafts in Vero cells before IBV attachment significantly reduced the expression of viral structural proteins, suggesting that drug treatment impaired the attachment of IBV to the cell surface. Our results indicated that lipid rafts serve as attachment factors during the early stages of IBV infection, especially during the attachment stage.

## 1. Introduction

Avian infectious bronchitis virus (IBV) is a member of the *Coronaviridae* family, which includes many human and animal pathogens of global concern, such as severe acute respiratory syndrome coronavirus (SARS-CoV), Middle East respiratory syndrome virus (MERS-CoV), and mouse hepatitis virus(MHV)[1]. IBV has a major economic impact on the global poultry industry because this prototype coronavirus considerably decreases hen egg production by impairing the upper respiratory and reproductive tracts of chickens [2]. IBV is an enveloped positive-stranded RNA virus with a 27–30 kb genome that encodes several polyproteins. The polyproteins of IBV are cleaved by viral proteases into at least 15 nonstructural proteins (NSPs) [3]. Four structural proteins, namely, spike protein (S), nucleocapsid protein (N), membrane protein (M), and small envelope protein (E), are encoded by subgenomic RNA species [4]. The binding and entry of IBV into host cells require interactions between cellular surface receptors and viral structural proteins that are involved in the attachment stage. Nearly all IBV field isolates can only be propagated in embryonated chicken eggs or transiently proliferated in primary chicken embryo kidney cells. However, the *Beaudette* strain, a cell-adapted strain, replicates efficiently in various cultured mammalian cell lines, including African green monkey kidney cells (Vero), human liver cancer cells, lung cancer cell lines, and baby hamster kidney cells through serial passages [5–8]. However, the detailed mechanism underlying virus-host interactions during viral attachment and entry remains uninvestigated. Here, we used the *Beaudette* strain as an *in vitro* model to study the mechanism of IBV infection.

As functional membrane microdomains, lipid rafts contain abundant cholesterol and sphingolipids [9], which are also termed detergent-insoluble glycolipid-enriched complexes or detergent-resistant membranes because of their significant features, such as composition and detergent resistance [10]. Specific microdomains exist in biological membranes; however, the existence of lipid rafts in living cells still remains controversial [11]. Lipid rafts are involved in some important cellular processes, including signal transduction, cell migration, and axonal guidance [12–15]. In addition, numerous studies have revealed that lipid rafts are important during viral infection. For example, lipid rafts are critical in multiple stages of the life cycles of dengue and hepatitis C viruses [16]. Furthermore, lipid rafts are involved in the binding and entry of host cells for several enveloped and non-enveloped viruses, including human immunodeficiency virus [17], poliovirus [18], human herpes virus 6 [19], West Nile virus [20], foot-and-mouth disease virus [21], and simian virus 40 [22]. Moreover, lipid rafts are involved in the assembly and egress of some viruses, including rotavirus, measles virus, Newcastle disease virus, influenza virus, Ebola virus, and Marburg virus [23–26].

Coronaviruses also require lipid rafts for cellular entry. Some studies showed that drug-mediated cholesterol depletion inhibited human coronavirus 229E and MHV entry into host cells [27,28]. In a previous study, we reported that lipid rafts are crucial for SARS–CoV entry into Vero E6 cells [29]. Moreover, changes in the lipid component of cellular membranes affect the outcome of IBV infection [30,31]. Intriguingly, the efficiency of IBV-induced membrane fusion considerably varies among different cell lines. In this study, we investigated the direct role of lipid rafts during IBV infection in Vero cells. Lipid rafts were disrupted by depleting cellular cholesterol with two pharmacological agents, methyl-β-cyclodextrin (MβCD) and Mevastatin. The results showed that the structural proteins of IBV are required for attachment to Vero cells. However, lipid rafts may not be involved in the replication and release of IBV. These results revealed that lipid rafts are required during the early stages of IBV infection.

## 2. Materials and Methods

### 2.1 Cells and viruses

Vero cells and DF1 cells were cultured in Dulbecco’s modified Eagle’s medium (DMEM, Life Technologies, Carlsbad, CA, USA) supplemented with 10% fetal bovine serum (FBS), penicillin (final concentration 100 U/mL), and streptomycin (final concentration 100 μg/mL). A549 cells were cultured in F-12K medium (Life Technologies) supplemented with 10% fetal bovine serum (FBS). All cells were purchased from American Type Culture Collection and incubated at 37°C in a 5% CO_2_ environment. IBV *Beaudette* strain [32] was passaged in Vero cells. Viral stocks were stored at -80°C prior to use. The 50% tissue culture infective doses (TCID_50_) of viral stocks were calculated by Reed–Muench method.

### 2.2 Chemicals and antibodies

MβCD and Mevastatin were purchased from Sigma-Aldrich (St. Louis, MO, USA). Anti-IBV structural proteins S, N, and M, as well as the non-structural protein NSP5 and NSP12 antibodies were obtained via immunization of rabbits [33]. Mouse monoclonal antibodies against human tubulin and β-actin were purchased from Sigma-Aldrich along with anti-raft-resident marker caveolin-1 and GM1 antibodies. Fluorescein isothiocyanate (FITC)-conjugated anti-mouse and anti-rabbit immunoglobulin G (IgG), as well as horseradish peroxidase (HRP)-conjugated anti-human, anti-mouse, and anti-rabbit IgG were purchased from Dako (Glostrup, Denmark).

### 2.3 Sucrose gradient fractionation for lipid rafts

Vero cells treated with or without drugs were incubated with IBV for 24 h at 37°C. The cells were scraped in ice-cold PBS and spun down at 20,656 g for 30 s at 4°C. The pellet was resuspended in 1 mL of TNEV buffer (150 mM NaCl, 25 mM Tris-HCl, pH 7.5, 5 mM EDTA, and 1% Triton X-100) containing a cocktail of protease and phosphatase inhibitors (Roche, Mannheim, Germany). The lysate was kept on ice for 30 min, and then homogenized with 20 strokes in a pre-chilled Dounce homogenizer. The supernatant was applied to a discontinuous sucrose gradient followed by centrifugation at 34,426 g for 15 min at 4°C. Then, the sucrose gradient was centrifuged at 55,000 g for 20 h at 4°C with SW40Ti rotor (Beckman, USA). Eleven fractions of 1 mL each were collected from top to bottom of the sucrose gradient solution and diluted in sodium dodecyl sulfate polyacrylamide gel electrophoresis (SDS-PAGE) loading buffer. Then, 10 μL of the samples were subjected to SDS-PAGE and Western blot analysis.

### 2.4 SDS-PAGE and Western blot analysis

Each sample was lysed in TNEV buffer containing a cocktail of protease and phosphatase inhibitors. Protein concentration was determined with Bio-Rad Protein Assay kit. Then, the lysate was added at the same volume as the 2× SDS sample loading buffer with bromophenol blue. Equal amounts of total protein were separated by SDS-PAGE and then transferred onto polyvinylidene difluoride membranes. The membranes were incubated with primary antibody for 1 h at 37°C, and then with HRP-conjugated secondary antibody for an additional 1 h at 37°C. The membranes were detected with ECL Advance Western Blot Detection Kit (Amersham, Marlborough, MA, USA).

### 2.5 Drug treatment of Vero cells

Vero cell monolayers were washed with serum-free medium. The cells were infected with IBV of 1 MOI for 1 h at 4°C before or after MβCD or Mevastatin treatment at 4°C for 1 h. Drug concentration was determined from the dose-response curve. The cells were washed twice with serum-free medium and then grown in serum-free medium for different periods. Cell culture supernatants and whole cells were harvested to detect viral genes or proteins by RT-PCR or Western blot analysis, respectively.

### 2.6 Analysis of viral RNAs by RT-PCR

Vero cells were seeded on 35-mm dishes and infected with 1 MOI IBV when the cell monolayer was approximately 90% confluent. The infected cells were treated with MβCD or Mevastatin for 1 h at 4°C and harvested at certain time points [0, 8, 12, 18, and 24 h post-infection (hpi)]. Total RNA was extracted, and the cDNA template was synthesized with PrimeScript^TM^ Reverse Transcriptase (Takara, Dalian, China). The target gene was amplified by PCR with synthesized cDNA and specific primer pairs. The primers used for amplification are listed in Table 1. The amplification program was set at 94°C for 25 s, 56°C for 25 s, and 72°C for 20 s for 20 cycles. The sizes and specificity of the PCR products were verified by agarose gel electrophoresis. The relative expression levels were calculated by the 2^-ΔΔCT^ method against the uninfected controls. GADPH gene was set as the endogenous control.

**Table 1:** Specific primers for S1 fragment and GADPH fragment.

Primer name	Primer sequences
S1 forward	5′-TGAGATTGAAAGCAACGCCAGTTG-3′
S1 reverse	5′- CTTACCATAACTAACATAAGGGC-3′
GAPDH forward	5′-CGGCATCCACGAAACTAC-3′
GAPDH reverse	5′-ATCTTCATCGTGCTGGGCG-3′

### 2.7 Immunofluorescence assay (IFA)

Vero cell monolayers that adhered to coverslips were transfected with plasmids encoding IBV E protein and were then incubated at 37°C for 8, 16, and 24 h. The cells were fixed with 4% paraformaldehyde (PFA) and treated with 0.1% Triton X-100. The cells were incubated with anti-IBV E protein or GM1 antibodies at 37°C for 1 h. Immunofluorescence confocal microscopy images were captured with LSM510 META microscope (Zeiss, Jena, Germany).

### 2.8 Densitometry and statistical analysis

The relative intensities of protein bands were analyzed with ImageJ software. Data were presented as mean ± standard deviation (SD). Results from different groups were compared by the Student’s *t* test. Values of p < 0.05 were considered statistically significant.

## 3. Results

### 3.1 Lipid rafts are associated with IBV structural proteins in IBV-infected vero cells

**Fig 1:**
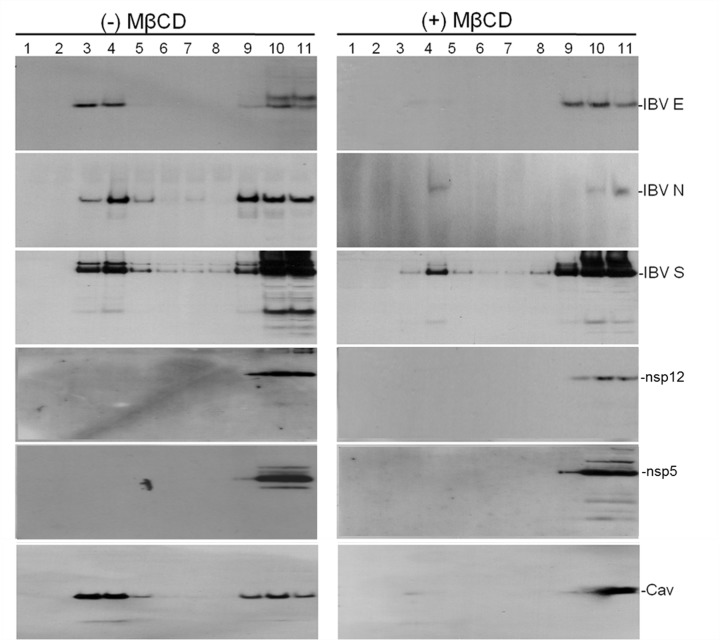
IBV structural proteins are associated with lipid rafts in IBV-infected Vero cells. Vero cells infected with IBV at 1 MOI for 24 h were lysed with TNEV buffer containing 1% Brij. Lysates were then analyzed by sucrose gradient centrifugation. Samples from each fraction were evaluated via Western blot analysis with specific antibodies against E, N, S, NSP5, and NSP12. Both lipid raft and non-lipid raft fractions were detected with cav-1.

Depending on their membrane locations, lipid rafts are mainly involved in viral entry and release. This study investigated the relationship between lipid rafts and IBV infection. Vero cells were infected with IBV *Beaudette* strain, and membrane flotation was performed by sucrose density gradient centrifugation after depleting plasma membrane cholesterol with MβCD. As shown in Fig 1, a significant proportion of the IBV structural proteins E, N, and S co-fractionated with caveolin-1 in fractions 3–5 without drug treatment. However, MβCD treatment considerably reduced the amounts of IBV structural proteins, including E, N, and S, in fractions 3–5. By contrast, the IBV NSP5 and NSP12 did not co-fractionate with the lipid rafts (Fig 1). The depletion of cholesterol via MβCD treatment did not affect the distribution of NSP5 and NSP12 in cellular fractions (Fig 1). In addition, N, E, S, and caveolin-1 shifted to fractions 10 and 11 after MβCD treatment. These results demonstrated that the structural proteins, and not NSPs of IBV, are partially associated with cholesterol-enriched raft domains in Vero cells during IBV infection.

### 3.2 IBV E protein co-localizes with GM1 and may translocate to the plasma membrane

**Fig 2:**
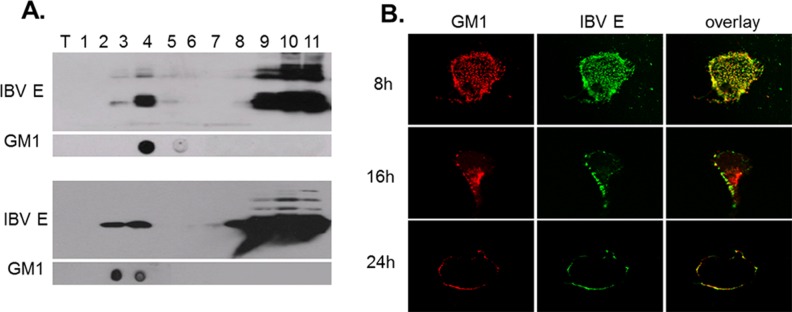
IBV E protein co-localizes with GM1. Vero cells were transfected with plasmids expressing IBV structural protein E and collected in TNEV buffer with 1% Brij at 16 h and 24 h after transfection. Lysates were analyzed by sucrose gradient ultracentrifugation. (A) Samples from each fraction were evaluated through Western blot analysis with specific antibodies against IBV E (above). The floating bands from sucrose gradient ultracentrifugation were collected and assessed by dot blot analysis with GM1 antibody (below). (B) Vero cells were transfected with plasmids encoding IBV E protein and fixed with 4% PFA at 8, 16, and 24 h post-transfection. The cells were then permeabilized and immunolabeled with IBV E and GM1 antibodies. Slides were analyzed via confocal laser scanning microscopy.

To further investigate the association of IBV structural proteins with lipid rafts, IBV E protein was overexpressed in Vero cells. The total cell lysates in sucrose gradients were analyzed by membrane flotation assay. The flotation gradient fractionations were monitored by Western blot analysis with specific antibodies against IBV E protein and assessed by dot blot analysis with the lipid raft marker ganglioside GM1 antibody. As shown in Fig 2A, IBV E protein floated up to gradient fractions 3–5 at 16 hpi. The ganglioside GM1 was also present in fraction 4. When transfection was extended to 24 h, IBV E protein floated in the gradient fractions 3 and 4, which are the same gradient fractions as GM1. This result suggested that IBV E protein can translocate with lipid rafts.

To directly observe the dynamic changes of co-localization and to verify the results of the flotation gradients, IFA was performed to investigate the interaction between IBV E protein and lipid rafts via confocal microscopy. Vero cells overexpressing IBV E protein were harvested for IFA detection at 8, 16, and 24 h post-transfection. IFA staining was performed with anti-IBV E and FITC-conjugated GM1 antibodies. As shown in Fig 2B, IBV E protein appeared both in the plasma membrane and cytoplasm at 8 h post-transfection and then gradually translocated to the plasma membrane. At 24 h post-transfection, IBV E protein was mainly distributed in the plasma membrane with GM1. The transfer between the intracellular and plasma membrane indicated that IBV E protein is functionally related to lipid rafts in a time-dependent manner after transfection. The results suggested that lipid rafts may be required for some stages of IBV infection.

### 3.3 Lipid rafts are not necessary for the release of IBV

**Fig 3:**
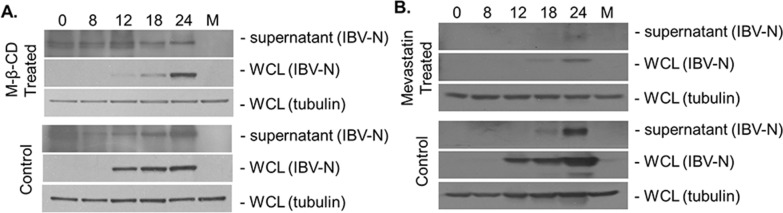
IBV structural proteins in the supernatant are unaffected by MβCD or Mevastatin treatment. Vero cells with or without drug treatment were infected with IBV at 1 MOI for 0, 8, 12, 18, and 24 h. Supernatants were directly collected and the cells were lysed for Western blot assay. The samples were evaluated with specific antibodies against the IBV N protein. Tubulin was used as loading control marker.

The co-translocation of IBV E protein with lipid rafts on the cellular membrane suggested that lipid rafts are involved in IBV infection. To investigate whether lipid rafts are involved in IBV release, cell supernatants and total lysates after IBV infection with or without MβCD treatment were collected to detect viral proteins with specific antibodies. As shown in Fig 3A, IBV N protein was detectable in whole cell lysates (WCL) until 12 hpi. Notably, the expression levels of IBV N protein in the WCL and the supernatants significantly decreased after MβCD treatment compared with the control, especially at 12–18 hpi in the WCL. Moreover, the ratio of IBV N protein in the WCL and supernatants was similar for treated and untreated infected cells. This result indicated that MβCD treatment did not influence the release of virions to the supernatant. Similarly, Mevastatin treatment also significantly and simultaneously decreased viral N protein levels both in the WCL and the supernatants. These results suggested that Mevastatin and MβCD affected IBV replication in the cytoplasm and reduced the number of viral particles released into the supernatant. Additionally, lipid raft disruption did not influence the release of virions into the supernatant. The results provided evidence that lipid rafts are required for the early stages of IBV infection before virion release stage.

### 3.4 Lipid rafts do not affect virus genome replication

**Fig 4:**
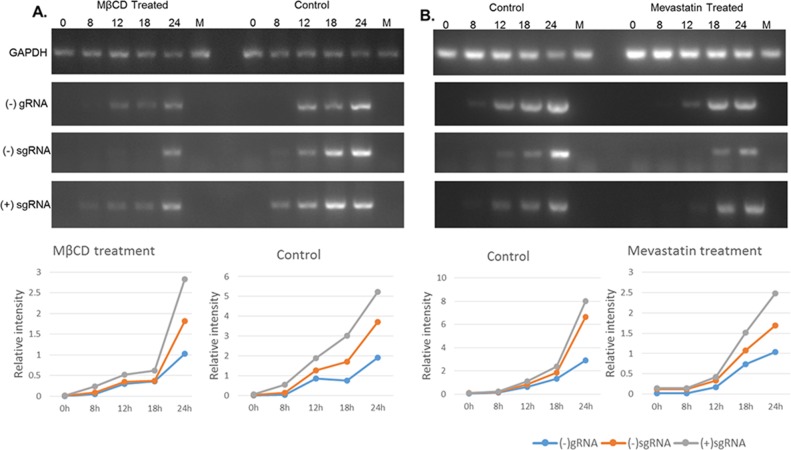
IBV genome replication is evaluated by RT-PCR assay after MβCD or mevastatin treatment. Drug-treated Vero cells after IBV infection at 1 MOI for 1 h at 37°C were collected at 0 h, 8 h, 12 h, 18 h, and 24 h. Total RNA was extracted from the cells. Negative-strand gRNA and subgenomic mRNA were detected by semi-quantitative RT-PCR. Line charts represent the tendency on the basis of PCR results.

Our previous study found that IBV localized with GM1 protein at different infection stages. To determine the effect of lipid rafts on IBV genome replication, Vero cells treated with MβCD or mevastatin after IBV infection were collected at different time points to evaluate the level of viral RNA copies. IBV RNA copies were determined by semi-quantitative RT-PCR with specific primers. The RNA level represents the IBV genome copies after IBV replication. Fig 4A and 4B show that RNA copies, including negative-strand genomic RNA (gRNA) and negative and positive subgenomic RNA (sgRNA) were evaluated by semi-quantitative RT-PCR. The results showed that the viral RNA levels increased over time after IBV infection. Notably, both gRNA and sgRNA exhibited rapid increases in RNA levels regardless of drug treatment, indicating that drug-induced lipid raft disruption could not suppress IBV viral genome replication. The RNA levels of drug-treated Vero cells decreased at each time point after IBV infection compared with the control, suggesting that drug treatment may affect IBV virion entry into cells during the early stages of infection. These results demonstrated that lipid rafts may function in the early stages of virion entry into cells, but do not affect viral genome replication.

### 3.5 Lipid rafts are involved in the attachment step of IBV endocytosis

**Fig 5:**
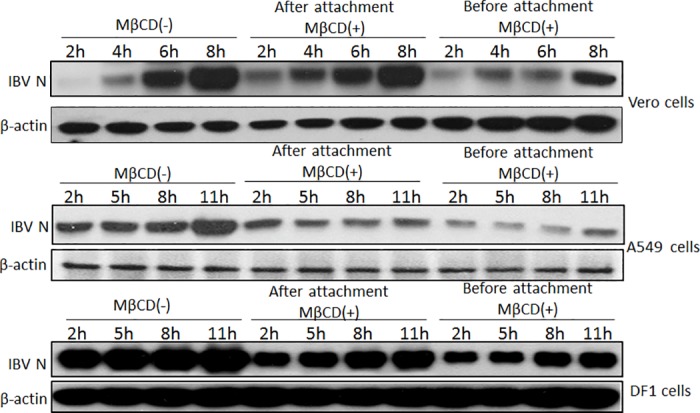
Lipid rafts are involved in virus attachment. Vero, A549, and DF1 cells were treated with MβCD before or after IBV infection at 10 MOI. Cells were incubated at 37°C for indicated time points. The cells were then lysed and analyzed by Western blot with specific antibodies against IBV N protein. β-actin was used as the loading control.

Lipid rafts are required for IBV infection, but are not involved in viral genome replication and particle release. To further study the participation of lipid rafts in the early stages of viral infection, as well as to identify the specific involvement of lipid rafts in viral endocytosis, lipid rafts were inactivated with MβCD. To determine the role of lipid rafts in IBV attachment, host cells were pre-treated with MβCD for 1 h at 4°C, incubated with IBV for 1 h at 4°C, and then incubated at 37°C for 2, 4, 6, and 8 h. To determine the role of lipid rafts in IBV entry, host cells were pre-incubated with IBV for 1 h at 4°C, treated with MβCD for 1 h at 4°C, and then cultured at 37°C for 2, 4, 6, and 8 h. Vero cells without the MβCD treatment were set as the control. To assess viral replication, IBV N protein was subjected to Western blot analysis. As shown in Fig 5A, IBV N was detectable in the control group with increasing hpi. MβCD treatment decreased IBV N levels compared with that of the control group. In particular, MβCD treatment prior to IBV attachment significantly repressed IBV N expression. This result suggested that MβCD treatment blocked viral particles from binding with the cell membrane. To further confirm the function of lipid rafts in IBV endocytosis, the IBV-susceptible cell lines A549 and DF1 were treated with MβCD before or after IBV attachment (Fig 5B and 5C). The depletion of lipid rafts by MβCD suppressed IBV N expression in both cell lines when cells were treated with MβCD before IBV attachment. However, this result was not observed when cells received MβCD treatment after IBV attachment. These results indicated that MβCD treatment directly inhibited IBV attachment and that drug-induced lipid raft disruption mainly blocked IBV attachment to host cells. Therefore, lipid rafts are crucial to virus endocytosis and are required for IBV attachment.

## 4. Discussion

Viral infection comprises several steps, and viral attachment to host cells is the first step that determines tissue tropism, host specificity, and viral pathogenicity [34]. Although some viruses can recognize numerous host species, other viruses have limited host ranges because of their requirement for specific host cell receptors. In addition to special receptors, lipid rafts, as membrane components that ubiquitously exist in all cell types [35], are also involved in the regulation of endocytosis, intracellular trafficking of growth factor receptors, and localization of endosomes in cells [36]. A growing number of studies in the past two decades have reported that lipid rafts are crucial in the pathogenesis of many viruses [37,38] and bacteria [39]. Moreover, lipid rafts are involved in different stages of the viral life cycle, such as viral entry and release.

IBV is a prototype avian coronavirus that belongs to the genus *Gamma- coronavirus*. It is an enveloped, positive-strand RNA virus. Its envelope contains the major attachment spike protein (S), the membrane protein (M), and the minor envelope protein (E). The spikes are composed of S protein trimers, which are necessary for viral attachment and subsequent fusion of viral and cellular membranes. Therefore, the IBV S protein and other structural proteins likely interact with lipid rafts. These direct or indirect interactions can affect the infection cycle of the virus. To test this hypothesis, we performed several experiments to determine the function of lipid rafts in IBV infection. Our data showed that IBV structural proteins co-localized with lipid rafts in the plasma membrane and that IBV structural proteins remain localized with the GM1 protein. In addition, the drug-induced disruption of lipid rafts in Vero cells inhibited IBV infection, which likely occurred because lipid rafts are involved in viral attachment. These results indicated that lipid rafts in the cellular surface may mediate viral adhesion to facilitate IBV endocytosis.

Although IBV receptor(s) remain unidentified[40], the association of IBV structural proteins with lipid rafts may increase the local concentration of viral structural proteins, particularly that of IBV S protein(Fig 1), which may facilitate the engagement of IBV S protein with its receptors and enhance IBV infection. Lipid rafts, as attachment factors, may promote viral entry into cells. Although our study confirmed the role of lipid raft in IBV infection, the fate of endocytosed IBV remains unclear. Previous studies found that IBV internalization is mediated via macropinocytosis, in which lipid rafts transit through early endosomes to the recycling endosomes and then back to the cell surface to form new adhesions [41,42]. Meanwhile, in micropinocytosis, pathogen-host interactions change the downstream signaling network to modify membrane traffic and cytoskeletal dynamics to meet each other’s requirements. It is likely that pathogen-host interactions promote lipid raft clustering and focal adhesion formation during endocytosis. The internalized IBV will also be transferred from early endosomes to later endosomes. Although the present study primarily focused on lipid rafts and its role in the early stages of viral infection, the significance of the involvement of lipid rafts in IBV entry goes beyond this molecule. Moreover, further studies should investigate whether protein molecules in lipid rafts serve as a signaling platform to activate downstream kinases and cellular adhesive formation.
